# Evidence for Dsg3 in regulating Src signaling by competing with it for binding to caveolin-1

**DOI:** 10.1016/j.dib.2015.11.049

**Published:** 2015-12-03

**Authors:** Hong Wan, Kuang Lin, Siu Man Tsang, Jutamas Uttagomol

**Affiliations:** a Queen Mary University of London, Barts and The London School of Medicine and Dentistry, Centre for Clinical and Diagnostic Oral Sciences, Institute of Dentistry, London; b Institute of Psychiatry, King׳s College London, London

**Keywords:** Desmoglein, Src signaling, Caveolin, Sequence alignment

## Abstract

This data article contains extended, complementary analysis related to the research articles entitled “Desmoglein 3, via an interaction with E-cadherin, is associated with activation of Src” (Tsang et al., 2010) [1] and figures related to the review article entitled “Desmoglein 3: a help or a hindrance in cancer progression?” (Brown et al., 2014) [2]. We show here that both Src and caveolin-1 (Cav-1) associate with Dsg3 in a non-ionic detergent soluble pool and that modulation of Dsg3 levels inversely alters the expression of Src in the Cav-1 complex. Furthermore, immunofluorescence analysis revealed a reduced colocalization of Cav-1/total Src in cells with overexpression of Dsg3 compared to control cells. In support, the sequence analysis has identified a region within the carboxyl-terminus of human Dsg3 for a likelihood of binding to the scaffolding domain of Cav-1, the known Src binding site in Cav-1, and this region is highly conserved across most of 18 species as well as within desmoglein family members. Based on these findings, we propose a working model that Dsg3 activates Src through competing with its inactive form for binding to Cav-1, thus leading to release of Src followed by its auto-activation.

**Specifications Table**

**Table:** 

Subject area	*Biology*
More specific subject area	*Cell signaling*
Type of data	*Western blotting, image, sequence alignment, graph*
How data was acquired	*Confocal microscope, co-immunoprecipitation, proximity ligation assay, bioinformatics*
Data format	*filtered, analyzed*
Experimental factors	*Cell culture, RNAi mediated knockdown of Dsg3, calcium depletion and repletion*
Experimental features	*in vitro analyses of protein-protein interaction and protein sequence alignment*
Data source location	*Original source of protein sequences used for alignment is from the NCBI Proteins*
Data accessibility	*Data is with this article*

**Value of the data**

• Dsg3 forms a complex with Src, caveolin-1 and E-cadherin.
• Dsg3 competes with Src for binding to caveolin-1 and thus elicits Src auto-activation.
• A highly conserved putative region within the carboxyl-terminus of Dsg3 is identified for binding to the scaffolding domain of caveolin-1.

## 1 Data

**Fig. 1:**
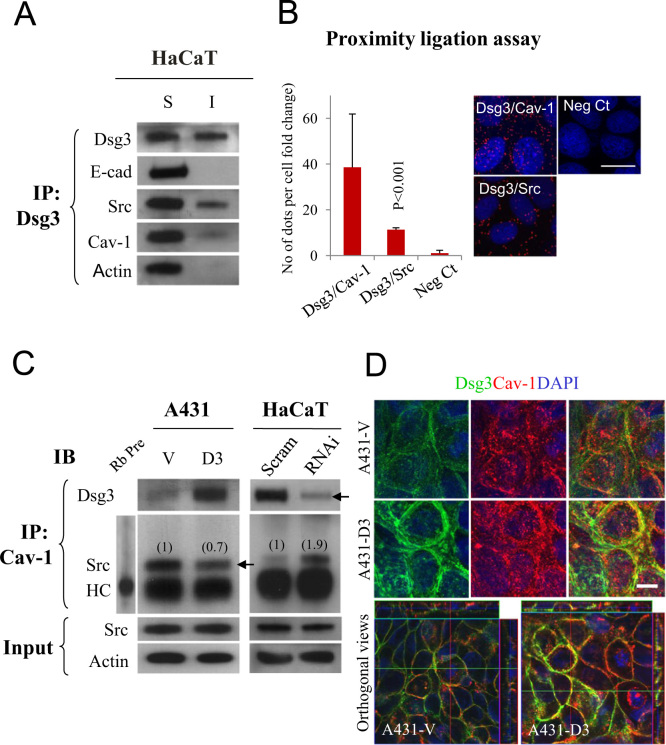
**Dsg3 competes with Src for binding to caveolin-1.** (A) Western blotting analysis of the Dsg3 immunoprecipitates from Triton-soluble and insoluble fractions of HaCaT keratinocytes and probed with the indicated antibodies. (B) Proximity ligation assay (PLA), left, for Dsg3 and Src or caveolin-1 (Cav-1) that showed the enhanced protein interaction signals for both Dsg3/Src and Dsg3/Cav-1 and the representative images of PLA are displayed on the right. (C) Western blotting of immune complexes purified with Cav-1 antibody from A431 and HaCaT cell lysates that showed the overexpression of Dsg3 resulted in a reduction of Src in the caveolin complex, and an inverse effect was observed in cells with Dsg3 silencing (RNAi), compared to the respective control cells (arrows). The densitometry of the Src was indicated above the Src blots. Scram: scrambled control siRNA, HC: antibody heavy chain. (D) Confocal microscopy showed enhanced colocalisation of Dsg3/Cav-1 in cells with overexpression of Dsg3 (A431-D3) compared to vector control cells (A431-V). Top panels are the projected images of the stacks (with maximum intensity) and bottom ones are the orthogonal views if stacks. Scale bar, 10 µm.

The desmosomal cadherin, Desmoglein 3 (Dsg3) is a calcium-dependent adhesion protein in epithelial cells and has recently been identified to associate with E-cadherin/Src and act as an upstream regulator in E-cadherin/Src signaling [1], [2], [3], [4], [5]. However, the molecular mechanism by which Dsg3 regulates Src remains poor understood. *In vitro* study showed that Dsg3 is internalized through a lipid raft-mediated pathway upon PV-IgG binding [6] and lipid raft contains caveolin protein. Interestingly, the Dsg3 associated family member Dsg2 is recently found to interact directly with the scaffold domain of caveolin-1 [7]. Hence, we speculated that Dsg3 also forms a complex with caveolin-1 along with Src. To investigate this possibility, we performed co-IP experiments with mouse antibody against Dsg3 in Triton-soluble and insoluble fractions of HaCaT cells, respectively, using the same procedures as previously described [1], [4]. Western blotting of immunoprecipitates revealed that both caveolin-1 and Src co-purified with Dsg3, alongside E-cadherin and actin, in particular from Triton-soluble fraction (Fig. 1A). The proximity ligation assay (PLA) showed that, compared with the negative control, there was a substantial increase of PLA signals in cells probed with either Dsg3/caveolin-1 or Dsg3/Src antibody combinations (Fig. 1B left bar chart) and Dsg3 silencing resulted in a reduced PLA signals as expected (data not shown).

Several lipid-regulated signaling molecules, including Src, Gα subunits and H-Ras, bind caveolin [8], [9]. Src of inactivated form is identified to bind to a membrane-anchored scaffolding domain of caveolin; the 20aa stretch within a membrane-proximal region of the cytosolic N-terminal domain of caveolin [8] (see cartoon in Fig. 5B). This 20aa residues functionally inhibit the auto-activation of c-Src and Fyn tyrosine kinases [8]. Hence, we hypothesized that Dsg3 may compete with inactive Src for the same binding site on caveolin. To test this hypothesis, we analyzed the immune complexes purified with caveolin-1 antibody in A431-Vect control and A431-hDsg3.myc cells with overexpression of Dsg3. Western blotting of caveolin-1 immunoprecipitates showed that overexpression of Dsg3 increased its association with caveolin-1 while reducing the amounts of Src in such a complex, compared to vector control cells (Fig. 1C left panels). In parallel, co-IP was performed in HaCaTs with or without Dsg3 depletion. Western blotting analysis of immunoprecipitates showed that Dsg3 silencing resulted in an increase in the amount of Src in the complex purified by caveolin-1 antibody (Fig. 1C right panels). Furthermore, confocal analysis indicated enhanced co-localization of Dsg3 and caveolin-1 at the plasma membrane in cells with overexpression of Dsg3 relative to vector control cells (Fig. 1D).

**Fig. 2:**
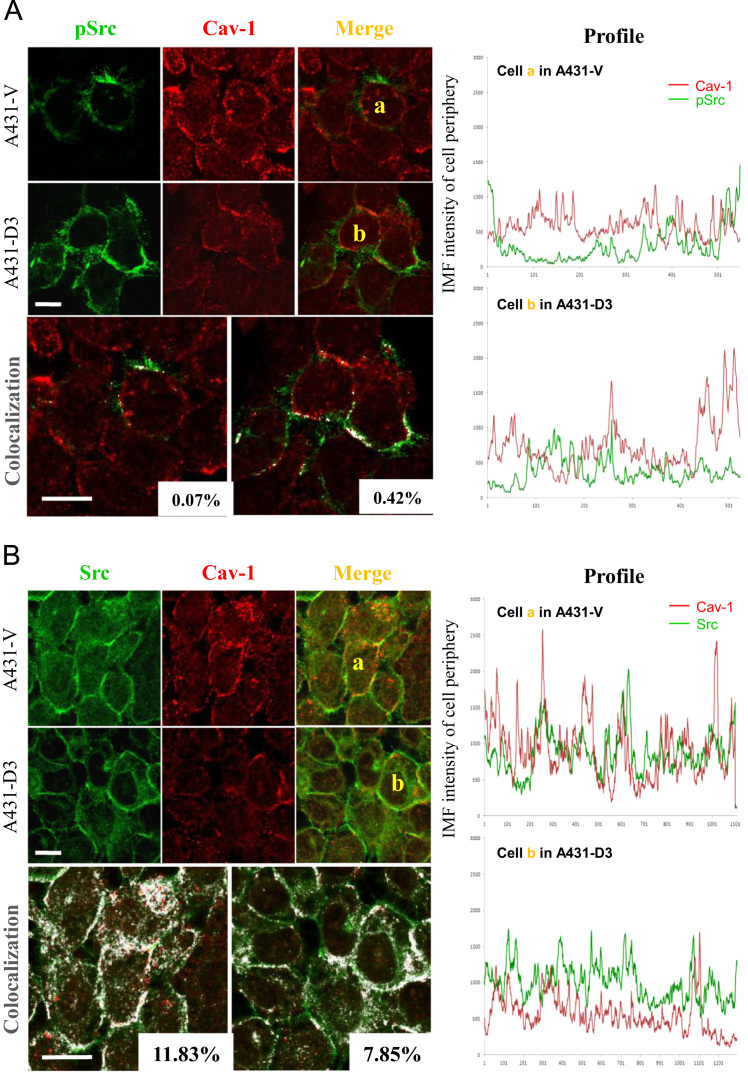
**The Dsg3 overexpressing cells showed reduced colocalization of caveolin-1 and total Src at cell periphery.** Immunofluorescence and colocalization analysis of caveolin-1 (Cav-1, red)/phospho-Src (pSrc, green) (A) and Cav-1 (red)/total Src (green) (B) in A431 cell lines. Cells were seeded at sub-confluent density for one day and fixed with ice cold Methanol for 10 min before proceeding immunofluorescence (IMF) staining. (A) Little colocalization of Cav-1/pSrc was detected at the cell periphery (highlighted in white pixels in the bottom two images). Phospho-Src was found predominantly expressed in the membrane protrusions and was not co-localized with Cav-1. Reduced expression of Cav-1 was observed in A431-D3 cells compared to A431-V control in which an enhanced Cav-1 staining at cell borders was noticeable. (B) A significantly enhanced colocalization of Cav-1/Src was seen in A431-V cells as compared with that for Cav-1/pSrc staining. The Dsg3 overexpressing cells (A431-D3) showed relatively less colocalization than that of A431-V cells for Cav-1/total Src staining (highlighted white pixels in the bottom two images). The profiles of IMF staining at cell borders for the marked cells (a and b) in A and B are displayed on the right, respectively. The percentage of colocalization in each image was indicated at the bottom right corner. Scale bar, 10 µm.

To test our hypothesis further, we performed double immunostaining with antibody combination for Cav-1/phospho-Src and Cav-1/total Src, respectively, followed by colocalization analysis with ImageJ. As shown in Fig. 2, there was little colocalization for Cav-1/pSrc at the cell borders in A431 cells and pSrc was predominantly expressed in the membrane protrusions. However, a marked increase in the colocalization of Cav-1 and total Src was detected at the cell borders in A431-V cells but this was found to be reduced in Dsg3 overexpressing cells (A431-D3) (see the colocalization images and profiles in Fig. 2B). Interestingly, a reduced expression level of Cav-1 was also observed in A431-D3 cells compared to A431-V control in which an enhanced Cav-1 staining at cell borders was noticeable.

**Fig. 3:**
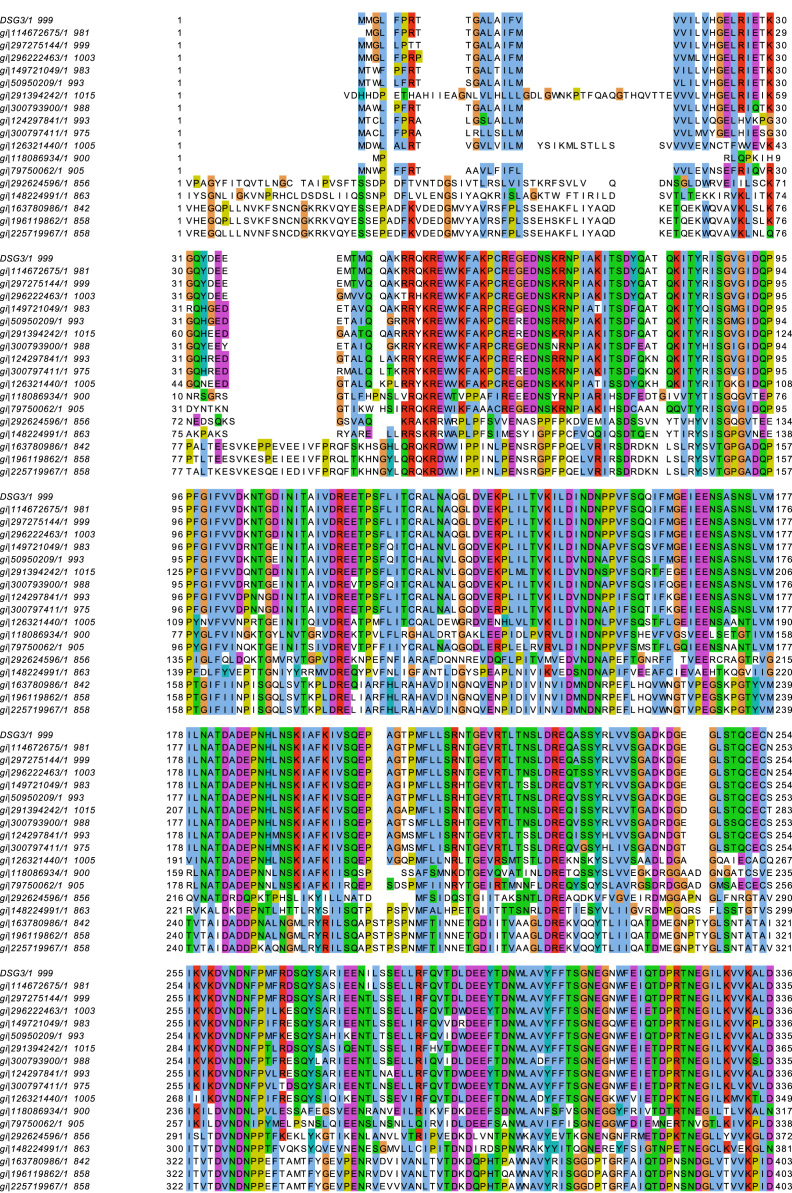

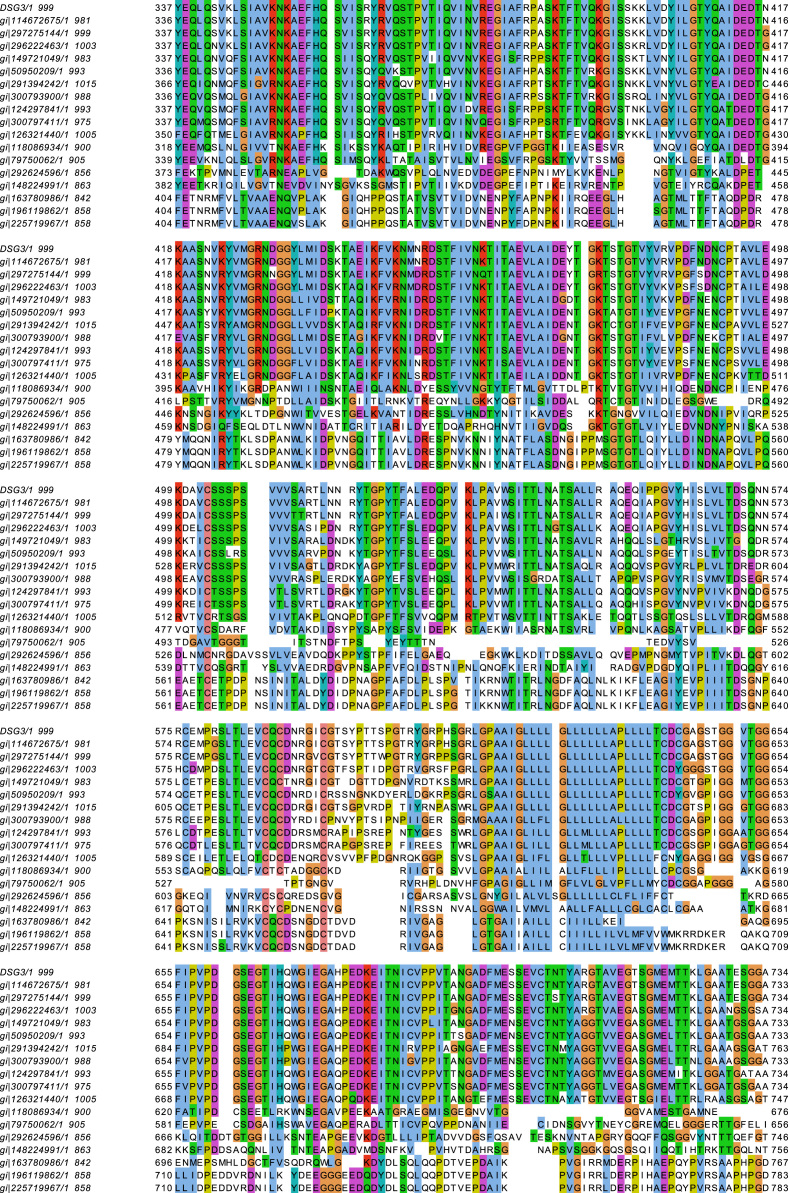

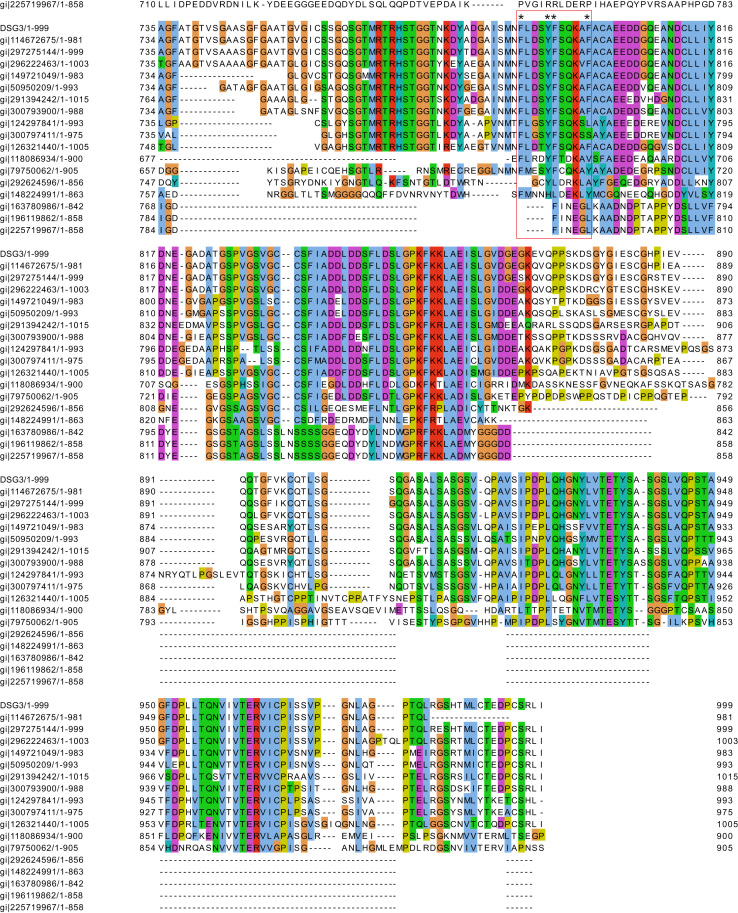
**A conserved putative binding site is identified within the carboxyl-terminus of Dsg3 by sequence alignment.** Alignment of Dsg3 across 18 species using the program MUSCLE [14]. The putative binding site (box with red line), at amino acid sequence between 788-798 with four aromatic amino acid residues (asterisks), for the scaffolding domain of caveolin-1 [10] is depicted to be conserved across 10 of 18 species. The amino acids are coloured according to the Clustal X Colouring Scheme [15].

**Fig. 4:**
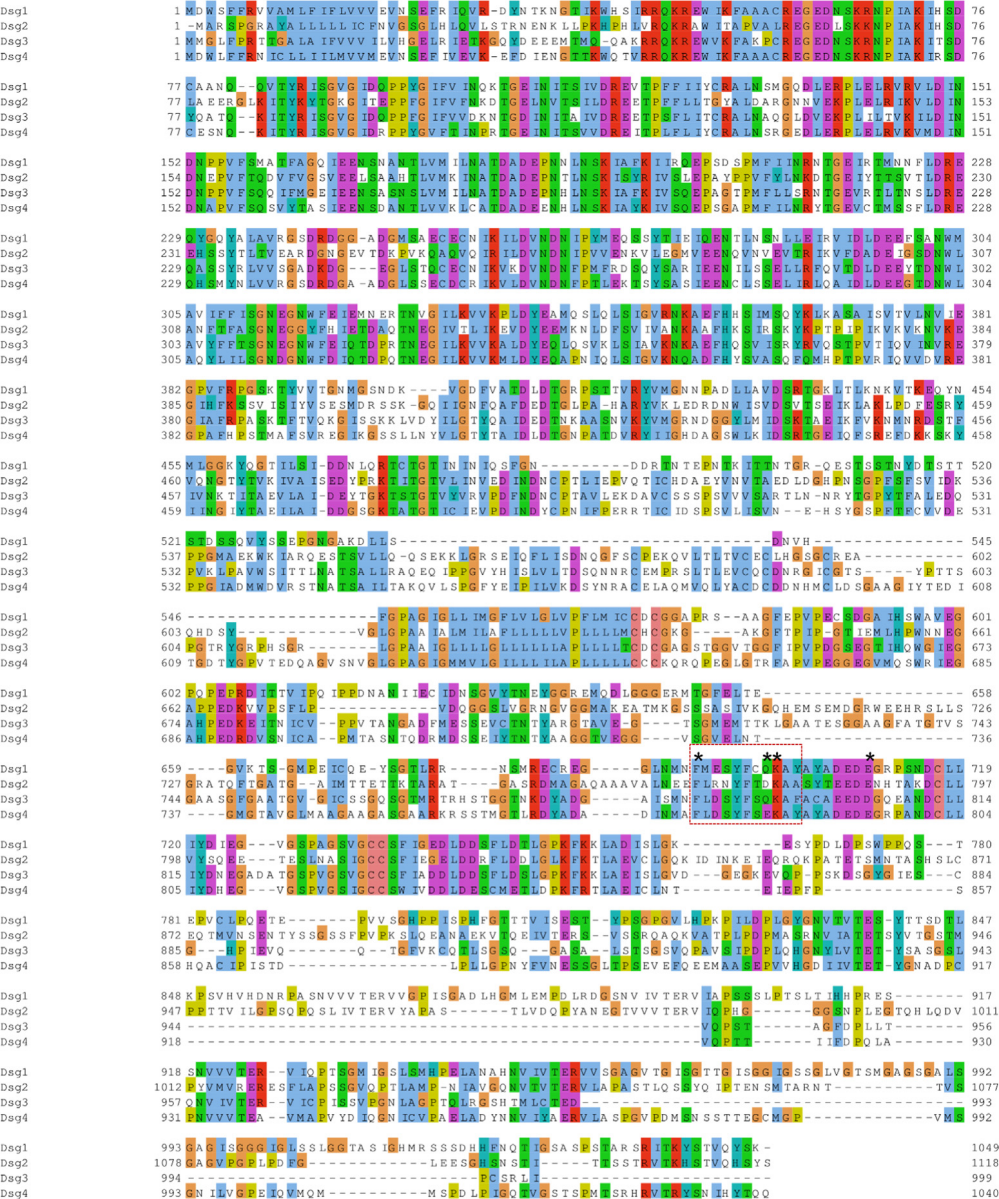
**The putative binding site is conserved within human Dsg subfamily.** The four family members of human Dsgs were aligned using the program MUSCLE [14]. The putative binding site for the scaffolding domain of caveolin [10] is highlighted in the box with a dotted line, in which four aromatic amino acid residues are depicted to be highly conserved across the family members (asterisks).

The protein sequence analysis identifies a putative region for binding the caviolin-1 scaffolding domain enriched in aromatic amino acids [10] in Dsg3 which is highly conserved across most of 18 species (Fig. 3) as well as within the Dsg family members (Fig. 4). This potential binding site is located within the ICS domain of C-terminus of Dsg3 at 788-798aa, an 11aa stretch which contains 4 aromatic aa residues and which likely competes with the inactive Src for binding to the scaffolding domain in caveolin-1 [10]. Interestingly, this region is overlapped with previously identified segment by Andi and Stanley [11], the 87aa sequence within the ICS domain downstream from the 779aa, that is required for binding to plakoglobin and recruitment of Dsg3 to desmosomes. Study based on plakoglobin null keratinocytes by Green and colleagues has suggested that plakoglobin plays a role to suppress Src activity [12]. Thus it is possible that when plakoglobin is ablated the excess free Dsg3 molecules compete with Src for binding the scaffolding domain of caveolin-1 thus elicits Src auto-activation. In accord, when Dsg3 is overexpressed the same result would occur with a consequence of Src activation.

**Fig. 5:**
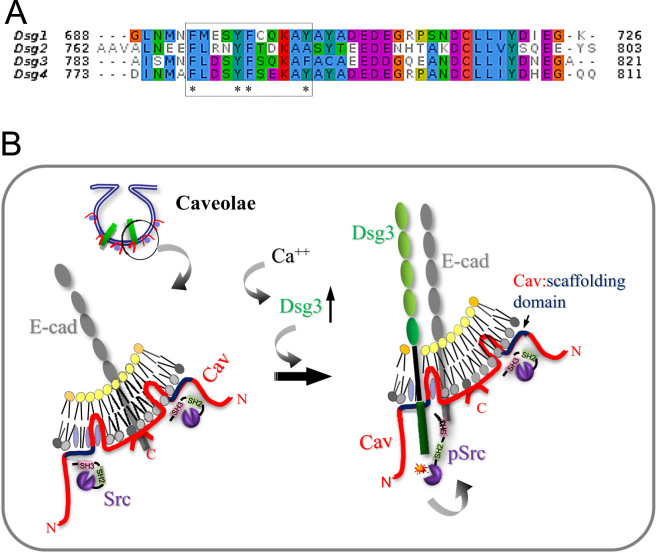
**A proposed working model of how Dsg3 activates Src.** (A) Amino acid sequence alignment of Dsg family members showing highly conserved putative region (dotted line) for binding to the scaffolding domain of caveolin-1 [7,10]. Asterisks indicate conserved aromatic amino acid residues across the family members. (B) Cartoon illustrating a possible mechanism by which Dsg3 regulates Src activity, *i.e.* expression and membrane distribution of Dsg3 induced by calcium renders its binding to Cav-1 within the caveolae and thus causes release of Src from its interaction with Cav-1, leading to auto-activation of Src that likely associates with E-cadherin (E-cad). Cav: caveolin-1, N: N-terminus of caveolin, C: C-terminus of caveolin, pSrc: phospho-Src.

Based on these findings we have proposed a working model that Dsg3 competes with inactive Src for binding to the scaffolding domain of caveolin-1, and thus causes Src to release from binding to caveolin-1, that leading to its auto-activation (Fig. 5B). This working model opens up a new avenue for future research.

## 2 Experimental design, materials and methods

### 2.1 Antibodies

The mouse monoclonal and rabbit polyclonal Abs used were: 5H10, mouse Ab against Dsg3 (Santa Cruz Biotechnology, Inc); HECD-1, mouse anti-E-cadherin (Abcam); Src (32G6) rabbit mAb and phospho-Src family (Tyr416) (Cell Signaling); rabbit caveolin-1 Ab (Cell Signaling); mouse mAb against caveolin-1 (Santa Cruz Biotechnology); rabbit anti beta actin-loading control ab8227 (Abcam); Secondary Abs were Alexa Fluor 488 conjugated goat anti-mouse IgG and Alexa Fluor 546 conjugated goat anti-rabbit IgG (Invitrogen).

### 2.2 Transient Dsg3 siRNA transfection

The siRNA sequence corresponding to nucleotides 620–640 of the respective coding region in human Dsg3 mRNA (Accession: NM_001944.1) (AAATGCCACAGATGCAGATGA) was used to knockdown Dsg3. The scrambled control siRNA was a randomized sequence of RNAi (AACGATGATACATGACACGAG). Both sequences were synthesized by Dharmacon Research (USA). The transfection procedures were described previously [13].

### 2.3 Co-immunoprecipitation (co-IP) and Western blotting

For analysis of protein in Triton X-100 -soluble and -insoluble fractions, cells were grown to freshly confluence in 100 mm Petri dishes prior to protein extraction in 500 µl of Trition X-100 lysis buffer (10 mM Tris–HCl, pH 7.5, 150 mM NaCl, 2 mM ethyleneglycol-bis-(β-aminoethylether)-N,N,N′,N′-tetraacetic acid (EGTA), 5 mM ethylenediamine tetraacetic acid (EDTA), 1%Triton X-100, 1 mM phenylmethylsufonyl fluoride and protease inhibitor cocktail (Boehringer Mannheim)) for 10 min at 4 °C. After centrifugation the supernatant was denoted as the Triton X-100 soluble fraction and the undissolved pellet subsequently was extracted in 200 µl RiPA buffer. After centrifugation the supernatant was collected and lysate was denoted as the Triton X-100 insoluble fraction. Protein concentration for each fraction was determined. 500 µg of protein in each fraction was used for co-IP experiment. For total cell lysate preparation, confluent cell cultures were washed with ice-cold PBS and lysed in ice cold RIPA buffer (Upstate) containing a protease-inhibitor cocktail (Calbiochem), for 10 min at 4 °C. Then lysates were clarified by microcentrifugation. The amount of 500 µg of total protein in each sample, as determined by *DC* protein assay (Bio-Rad), was used for IP. The rabbit caveolin antibody were coupled with Dynabeads (Invitrogen) for 3 h before addition into each lysate and incubated overnight at 4 °C with rotation. Immunoprecipitates were washed thoroughly prior to resuspension in 2× Laemmli sample buffer and boiled for 3 min. Aliquots of the denatured proteins were separated by SDS-PAGE and processed for Western blotting.

### 2.4 Duolink in situ proximity ligation assay for protein:protein interactions

Duolink proximity ligation assay (PLA) kit, composed of anti-mouse PLA probe plus, anti-rabbit PLA probe minus and detection kit 563, was purchased from Olink Bioscience (Cambridge Bioscience, UK). PLA assay was conducted following the protocol described with the kit. Briefly, cells grown on coverslips were fixed and then blocked with 10% goat serum (Sigma) for 15 min followed by incubation with primary antibody for 1 h at 37 °C. After this, coverslips were subjected to sequential incubation with PLA (plus+minus) probes for 2 h, hybridization for 15 min, ligation with ligase for 15 min, amplification with polymerases for 90 min and finally detection for 1 h. All these incubations were carried out at 37 °C and the coverslips were washed with 1×TBS-T under gentle agitation for 2–5 min between each incubation step. Finally, the samples were mounted and analyzed using a Leica DM5000 epi-fluorescence microscope. The negative control in this assay was A431-V cells labeled with mouse Dsg3 Ab (5H10) and rabbit anti-Myc tag Ab. The experimental samples were HaCaT cells labeled with mouse Dsg3 (5H10) and rabbit Cav-1 Ab or rabbit anti-Src, respectively. Statistical analysis was performed with Student׳s *t*-test and *p*<0.05 was considered statistically significant.

### 2.5 Immunofluorescence, confocal microscopy and colocalization analysis

For confocal analysis, cells were seeded at confluent or sub-confluent density on coverslips for one day. Then, cells were fixed with ice cold methanol and immune-fluorescently labeled using the mouse Dsg3 Ab (5H10)/ rabbit anti-caveolin-1 (in Fig. 1D) or the indicated antibody combinations (in Fig. 2). Coverslips were blocked with 10% goat serum for 20 min before the primary antibody incubation for 1 h at room temperature, followed by three washes for 5 min in PBS with 0.2% Tween 20. The secondary antibodies were Alexa 488 mouse IgG/ Alexa 546 rabbit IgG (Fig. 1D) or Alexa 488 rabbit IgG/ Alexa 546 mouse IgG (Fig. 2) and all were incubated for 1 h at room temperature. After two washes, coverslips were counterstained with DAPI for 10 min before a final wash and then mounted on slide. All antibodies were diluted at 1:100 in blocking buffer. Image stacks were acquired with Zeiss510 confocal microscope and analyzed with ImageJ for image processing and colocalization of double stained proteins. The presented images in Fig. 1D were Z projection with maximum intensity and in Fig. 2 were single confocal slice acquired at the highest protein expressing level in each region. The Colocalization Finder tool was used for colocalization analysis and the profiles of IMF staining at the cell borders were analyzed with a Segmented Lines tool with a line width of 20 pixels. All graphs were plotted in Excel.

### 2.6 Protein sequence alignment

The protein sequence alignment analysis was performed using the MUSCLE program [14]. The amino acids are colored according to the Clustal X Coloring Scheme [15].
